# Bronchoscopic Laser Resection as a Monotherapy for Bronchial Carcinoid

**DOI:** 10.7759/cureus.54635

**Published:** 2024-02-21

**Authors:** David Tchkonia, Teona Mskhaladze, Vera Nemsadze, Nino Khartishvili, Tornike Jashi

**Affiliations:** 1 Pulmonology, European University, Tbilisi, GEO; 2 Pulmonology, Tbilisi State Medical University, Tbilisi, GEO; 3 Medical School, David Tvildiani Medical University, Tbilisi, GEO; 4 Medical School, Tbilisi State Medical University, Tbilisi, GEO

**Keywords:** obstruction, neuroendocrine, minimally invasive, bronchoscopic laser therapy, bronchial carcinoid

## Abstract

Lung carcinoid tumors are rare neuroendocrine cancers that primarily affect younger individuals and exhibit unique epidemiological characteristics unrelated to smoking or environmental factors. Symptoms may include coughing and wheezing. Bronchoscopic biopsy is the standard for diagnosis, with surgical resection as the gold standard treatment. Recent advances, such as laser resection, offer alternative options. We present a case of a 46-year-old female with bronchial carcinoid successfully treated using bronchoscopic diode laser therapy, highlighting its minimally invasive benefits. The success of this approach depends on tumor location, underscoring the importance of considering tumor characteristics in treatment decisions. Our report contributes to the evolving landscape of bronchogenic tumor management, emphasizing the need for continued research.

## Introduction

Lung carcinoid tumors, composed of neuroendocrine cells, are rare, constituting only 1-2% of all lung cancers, primarily affecting younger individuals. Unlike other lung cancers, their etiology remains unclear and unrelated to smoking or environmental pollutants. These tumors exhibit ethnic and gender disparities, with a higher prevalence among White individuals and women. Interestingly, a significant proportion of cases are asymptomatic, often detected incidentally. Symptoms, when present, typically include coughing or wheezing and, in cases of severe bronchial obstruction, may lead to respiratory complications or post-obstructive pneumonia [1].

Bronchoscopic evaluation with biopsy is the gold standard diagnostic method for pulmonary carcinoids [2]. While surgical resection remains the mainstay of treatment, advances in bronchoscopic techniques, including laser resection, have expanded therapeutic options. Laser resection, a minimally invasive and precise thermal ablation technique, offers immediate relief for central airway obstruction (CAO) from malignant or nonmalignant causes [3-6]. It is a valuable palliative measure when primary treatments like surgery or radiation therapy are impractical, also serving as an adjunct before salvage therapies. Laser resection is particularly effective for shorter tumors (<4 cm) with significant intraluminal components, often complementing other bronchoscopic or systemic therapies [7-13]. Unless the tumor is completely intraluminal, this approach is less than ideal for curative intent treatment [14].

This report presents a bronchial carcinoid case, highlighting its clinical presentation, diagnostic process, and the use of bronchoscopic laser resection. Through this case, we underscore the evolving landscape of bronchial carcinoid management, emphasizing tailored, innovative approaches for this unique pulmonary neoplasm.

## Case presentation

A 46-year-old female with no history of smoking was referred to our hospital due to a three-month history of progressive dyspnea, particularly exacerbated during physical exertion, as well as persistent fatigue. Despite prior treatment with broad-spectrum antibiotics and various bronchodilators, the patient's symptoms remained unresolved. Physical examination revealed a localized wheeze in the right upper lobe on auscultation, with oxygen saturation at 95% and a respiratory rate of 20 breaths per minute.

**Figure 1:**
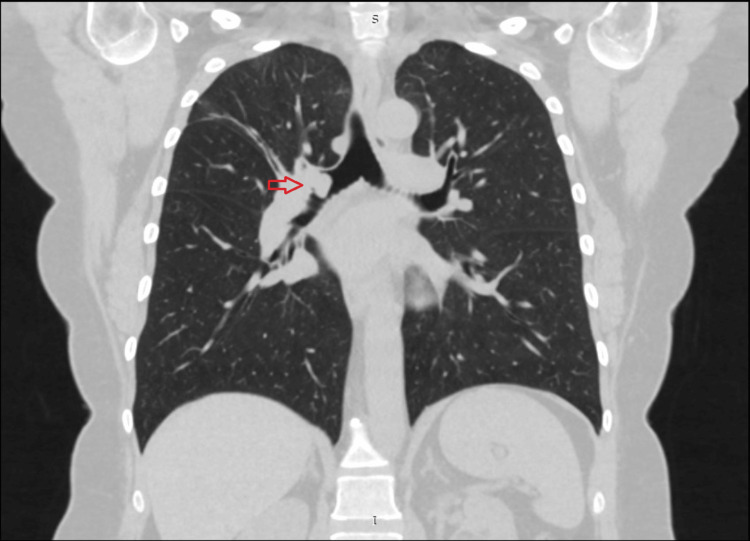
Diagnostic CT scan showing intraluminal mass (arrow)

**Figure 2:**
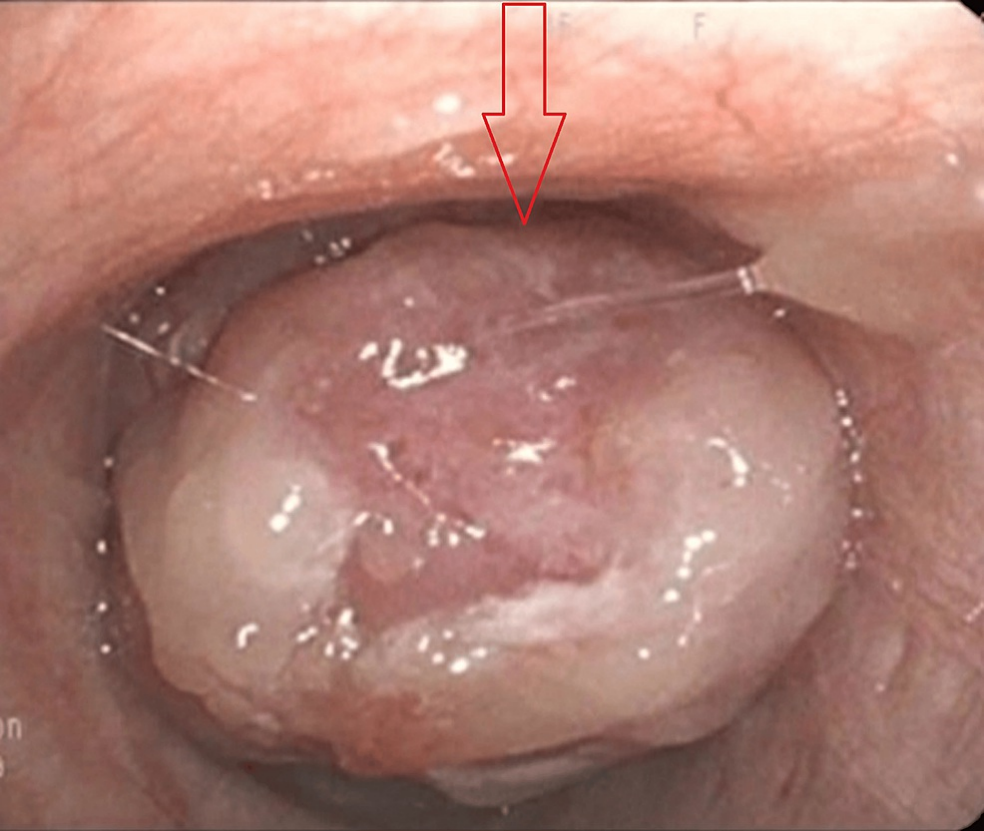
Bronchoscopic visualization of the intraluminal mass (arrow)

Two days later, a CT scan identified a 0.8 cm pedunculated mass within the right main and right upper bronchus, accompanied by atelectatic changes in the adjacent segment (Figure 1). There were no signs of metastases. Subsequently, a fibrobronchoscopic examination was conducted after a week, revealing complete obstruction of the right upper lobe bronchus by the aforementioned mass, with partial involvement of the main bronchus (Figure 2). Histopathological analysis of the resected material strongly indicated a highly differentiated, low-grade (G1) neuroendocrine tumor with low mitotic activity consistent with typical carcinoid pathology. Additional immunohistochemical studies confirmed the tumor's type and low grade, with Ki67 (1%), synaptophysin (100%), and CD56 (neural cell adhesion molecule (NCAM), 100%) positivity [15].

A month after the presentation, following thorough discussions among the team, which included interventional pulmonologists, a thoracic surgeon, and an oncologist, a bronchoscopic diode laser procedure was carried out to remove the mass. While surgery was considered an alternative option, the patient opted for laser resection to avoid a substantial loss of lung tissue. Initially, a low-voltage diode laser (7-8 watts) was used to treat the mass in the right main bronchus. It was then completely excised with a high-voltage diode laser (14-16 watts) and removed with forceps. The remaining tumor obstructing the right upper bronchus was resected using a high-voltage diode laser (16 watts) and also removed with forceps. During the procedure, a significant accumulation of purulent material was found in the right upper bronchus. To address this, we performed endobronchial lavage using 300 mL of normal saline. However, due to moderate bleeding, the lavage was conducted at a reduced rate, and 30 mL of tranexamic acid was locally administered to control the bleeding. The bleeding stopped, and no other complications arose. The patient was discharged the following day on prophylactic antibiotics, including ceftriaxone and azithromycin. The culture of bronchoalveolar lavage fluid detected *Staphylococcus aureus* sensitive to ceftriaxone, and the antibiotic regimen was not changed. The resected mass was rechecked through histopathology, confirming the diagnosis of bronchial carcinoid.

**Figure 3:**
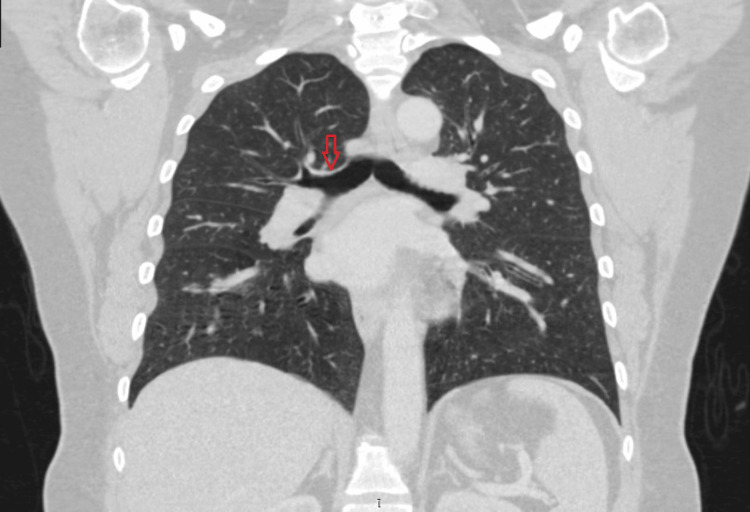
Postprocedural CT scan Arrow pointing towards the previous tumor location.

**Figure 4:**
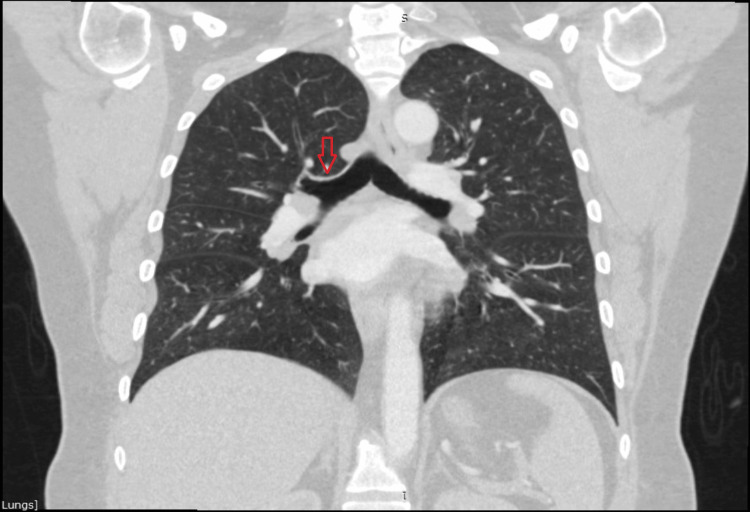
Follow-up CT scan Arrow pointing towards the previous tumor location.

**Figure 5:**
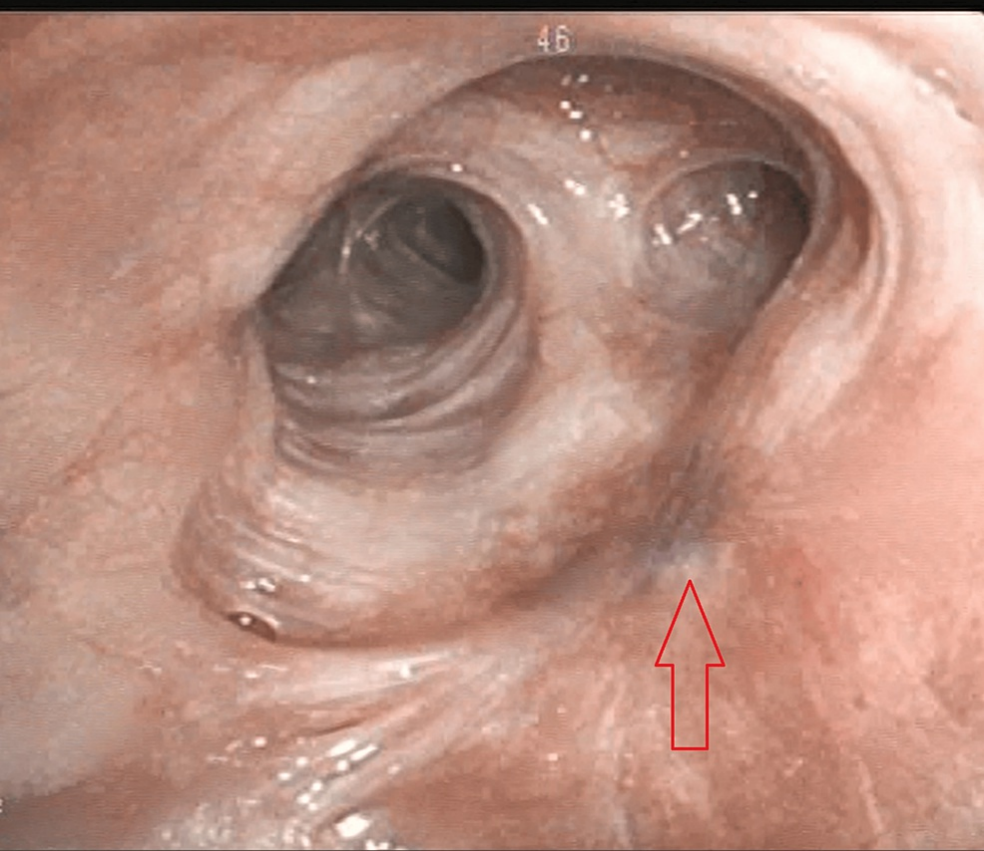
Follow-up bronchoscopy Arrow pointing towards the previous tumor location.

A CT scan was conducted two days after the laser resection (Figure 3) and repeated a year later (Figure 4), coinciding with the bronchoscopy (Figure 5) as part of the nine-month follow-up. Once again, there were no signs of metastases. The results were satisfactory, and the patient remained asymptomatic throughout this period.

## Discussion

This case report presents a unique perspective on the treatment of bronchial carcinoid tumors, particularly the application of bronchoscopic laser therapy as the primary treatment method. While surgical resection remains the standard approach for these tumors, our report highlights the potential of bronchoscopic laser therapy in managing appropriate cases.

Bronchoscopic laser therapy offers several advantages over traditional surgery, including quicker recovery and shorter hospital stays. A recent meta-analysis conducted by Reuling et al. highlighted that, as of the current state of research, no randomized trials have been conducted to directly compare bronchoscopic management in isolation with surgical treatment for pulmonary carcinoid tumors [16]. This is probably due to concerns about potential complications such as uncontrollable bleeding, bronchial stenosis, or vocal cord paralysis, in addition to a similar side effects profile as any other endobronchial procedure. Most importantly, there is the risk of late recurrent disease surpassing the scope of curative surgical intervention [16,17].

It is crucial to consider tumor characteristics when choosing the most appropriate treatment modality. Our successful procedure was largely dependent on the tumor's size and location, as it was small, entirely intraluminal, and relatively proximal to the trachea. Studies investigating prognostic factors for primary bronchoscopic management concluded that small intraluminal tumors measuring ≤2 cm without signs of extraluminal growth were best suited for endobronchial resection. In contrast, all tumors larger than 2 cm should be referred to surgery [16,17].

While this case highlights the potential of bronchoscopic laser therapy, it's important to acknowledge the possibility of tumor relapse, as evidenced by a prospective study showing a 7.8% recurrence rate in the initial bronchoscopic treatment group [17]. The low-grade nature of the tumor may offer hope for long-term remission, but further surveillance is required.

## Conclusions

Our case provides valuable insights into the treatment of bronchial carcinoid tumors, highlighting the potential of bronchoscopic laser therapy, and emphasizing the importance of tumor size and location. While we can draw on related research in the broader field of bronchoscopic interventions, further studies are needed to solidify the role of laser therapy as a reliable monotherapy option for this rare condition. The ongoing research in this area will continue to shape the evolving landscape of bronchogenic tumor management.
